# Introductory Address, Delivered in the Theatre of Geneva Medical College, December 1, 1841

**Published:** 1842-01-08

**Authors:** 


### BIBLIOGRAPHICAL NOTICES.

*Introductory address, delivered in the Theatre of Geneva Medical College, December* 1, 1841. By Frank H. Hamilton, M. D., Professor of the Theory and Practice of Surgery in Geneva Medical College; Pittsfield Medical College, Mass.; Castleton Medical College, and Woodstock Medical College, Vermont. Published by the Class. pp. 18.

Our limits allow us little space for noticing Introductories hereafter, but as these productions inform us of many important facts in relation to the existing state of medical instruction in this country, we must be permitted to employ them sometimes as texts for discourses wandering far from their immediate subjects, and as hooks on which to suspend remarks having no personal bearing upon their authors. In commenting upon the interests and organization of the whole profession, we have been, and probably shall be hereafter suspected of aiming at individual men or institutions. The suspicion shall never find any foundation in our feelings.

Dr. Hamilton, in his note to the committee of publication expresses a fear that the public will be less lenient than his class, to the faults and imperfections growing out of the unfinished state of the article. We shall not prove so; for it is unreasonable to expect great care in thought and style to be bestowed on mere introductories which are not designed to produce a permanent and wide-spread influence on medical opinion. Two points only, in the body of the discourse, interest us considerably. Judging by the context, Dr. H. is evidently no advocate of the results of "the *cacoethes secandi*," and it is pleasant to observe the diffusion of a just hostility to that child of certain unfortunate blunders in the organization of the really noble French school of surgery—the spirit of hyper-operation;—a spirit which has caused such an incalculable amount of mutilation and suffering within the last five years in France and Germany, and even in this country. We confidently anticipate the speedy subsidence of the epidemic *cacoethes*, but even the leniency due to the essay before us on grounds already stated, will not excuse us in passing by, without expostulation, the harshness and asperity of some of the phrases employed by the writer, in alluding to this subject. The medical profession cannot be effectively addressed like a political town-meeting, in rude or decidedly uncourteous terms; and no man, however elevated in station, can avoid the loss of influence when, in medical discussion, he allows even just indignation to become elevated into the semblance of anger. In defence of the justice of this censure we quote the following passage:

"Dieffenbach has since renounced, by a public declaration, this useless, and often bloody operation, for stammering; and his silly apes must, foresooth, renounce it also, or find themselves in the disagreeable attitude of pedlars, who are endeavoring to vend their miserable wares, while the manufacturer himself is present, declaring them a hoax. Had these men expended one sensible thought upon this subject, they might have saved themselves. from so ridiculous a dilemma.

"But it is not in this operation alone that surgeons have shown more 'veneration' and 'destructiveness' than 'common sense' and 'cautiousness,' or even 'conscientiousness.' We shall, in the course of these lectures, have occasion to mention others, and speak further of those men who are seized with a *cacoethes secandi*, and who would pine and die, if not furnished with a daily supply of blood. Detestable bloodsuckers! Do they hope to be ranked as surgeons, or men, in this enlightened day of humanity? Their brutal ambition is worthy only a Nero or a Caligula. We commend their memories to a like desecration."

Another feature in the lecture is equally gratifying, as evincing the increasing prevalence of the conviction that the term of study in collegiate schools is too short to be available in teaching properly even the elements of medicine. On this subject we will quote a few lines of the address:

"But if, young gentlemen, I have not found cause to complain of the means afforded me here for the illustration of my branch, I do complain of the limited time allotted to my department, as well as to all the other departments in this and other American schools. We occupy as much time in our several courses as is occupied upon the same subjects in any other institution this side of the Atlantic, and more than is occupied by many of the most respectable country schools. We have also the usual quota of professors, and attempt to teach all the branches of medical science. But surely it is a miserable economy of time, that we are allowed but four months to expound to you all of medicine and surgery, and you must have minds ready and comprehensive beyond a parallel to apprehend and retain all their subtleties. What magnus Apollo has ever discussed and demonstrated thoroughly, in eighty or ninety lessons, the principles and practice of surgery?"

It must be peculiarly painful for a teacher maintaining the opinions of the lecturer, to find himself dragged along by a system which permits and renders advisable the assumption of the manifold professional duties which devolve upon him as the simultaneous holder of the surgical chairs in four distinct medical colleges located within the bounds of three separate sovereignties. He will join with us, we feel assured, in the good-humored smile which is irresistibly awakened when we compare the immutable limits of the sun's annual revolution, with the time required for the exercise of such multifarious duties.

